# Influence of dams on sauger population structure and hybridization with introduced walleye

**DOI:** 10.1002/ece3.11706

**Published:** 2024-07-21

**Authors:** William C. Rosenthal, Elizabeth G. Mandeville, Ashleigh M. Pilkerton, Paul C. Gerrity, Joseph A. Skorupski, Annika W. Walters, Catherine E. Wagner

**Affiliations:** ^1^ Department of Botany University of Wyoming Laramie Wyoming USA; ^2^ Program in Ecology University of Wyoming Laramie Wyoming USA; ^3^ Department of Integrative Biology University of Guelph Guelph Ontario Canada; ^4^ Department of Zoology and Physiology University of Wyoming Laramie Wyoming USA; ^5^ Wyoming Cooperative Fish and Wildlife Research Unit University of Wyoming Laramie Wyoming USA; ^6^ Wyoming Game and Fish Department Cheyenne Wyoming USA; ^7^ U.S. Geological Survey Reston Virginia USA

**Keywords:** conservation genomics, dams, hybridization, reservoir, sauger, walleye

## Abstract

Dams have negatively affected freshwater biodiversity throughout the world. These negative effects tend to be exacerbated for aquatic taxa with migratory life histories, and for taxa whose habitat is fundamentally altered by the formation of large reservoirs. Sauger (*Sander candadensis*; Percidae), large‐bodied migratory fishes native to North America, have seen population declines over much of the species' range, and dams are often implicated for their role in blocking access to spawning habitat and otherwise negatively affecting river habitat. Furthermore, hybridization appears to be more frequent between sauger and walleye in the reservoirs formed by large dams. In this study, we examine the role of dams in altering sauger population connectivity and facilitating hybridization with introduced walleye in Wyoming's Wind River and Bighorn River systems. We collected genomic data from individuals sampled over a large spatial scale and replicated sampling throughout the spawning season, with the intent to capture potential variation in hybridization prevalence or genomic divergence between sauger with different life histories. The timing of sampling was not related to hybridization prevalence or population divergence, suggesting limited genetic differences between sauger spawning in different time and places. Overall, there was limited hybridization detected, however, hybridization was most prevalent in Boysen Reservoir (a large impounded section of the Wind River). Dams in the lower Wind River and upper Bighorn River were associated with population divergence between sauger upstream and downstream of the dams, and demographic models suggest that this divergence has occurred in concordance with the construction of the dam. Sauger upstream of the dams exhibited substantially lower estimates of genetic diversity, which implies that disrupted connectivity between Wind River and Bighorn River sauger populations may already be causing negative demographic effects. This research points towards the importance of considering the evolutionary consequences of dams on fish populations in addition to the threats they pose to population persistence.

## Graphical abstract

In this study, we examine the role of dams in altering sauger population connectivity and facilitating hybridization with introduced walleye in Wyoming's Wind River and Bighorn River systems. Overall, there was limited hybridization detected, however, hybridization was most prevalent in Boysen Reservoir (a large impounded section of the Wind River). Dams in the lower Wind River and upper Bighorn River were associated with population divergence between sauger upstream and downstream of the dams, and demographic models suggest that this divergence has occurred in concordance with the construction of the dam.

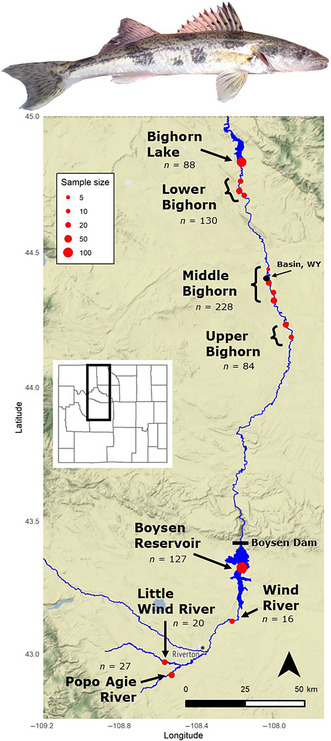

## 1 INTRODUCTION

Human landscape alterations provide important resources to people worldwide, but also have negative consequences on the health of aquatic ecosystems. Therefore, the balance between ecosystem service exploitation and preservation is complicated and important to consider (Dudgeon et al., 2006; Reid et al., 2019). The widespread construction of dams and other diversions is perhaps the most dramatic example of human activity affecting aquatic systems; there are over 75,000 dams in the United States alone that enable the storage of approximately 1 year worth of runoff (Graf, 1999).

Despite the benefits dams and diversions provide to food and energy production, they also alter natural hydrologic regimes and impede organism dispersal (Nilsson et al., 2005; Poff et al., 1997). The effects of dams on migratory fish populations are particularly well‐documented. Dams may block access to spawning habitat (Auer, 1996; Neraas & Spruell, 2001; Zhong & Power, 1996), alter thermal regimes during migration and spawning (Angilletta et al., 2008; Distefano et al., 1997; Zhong & Power, 1996), or prevent necessary larval fish drift (Humphries & Lake, 2000; Marotz & Lorang, 2018). However, the longer‐term evolutionary consequences of dam construction are not as thoroughly understood.

The effects of dams on migratory fish evolution are likely mediated by altering habitat and population connectivity. Changes in hydrologic and thermal regimes may impose selective pressures on juvenile and spawning adult fish (Angilletta et al., 2008) or increase ecological overlap between closely related species (Billington et al., 1997; Gangl et al., 2000; Rawson & Scholl, 1978). Blocking gene flow between previously connected populations can lead to population genetic divergence (Baumsteiger & Aguilar, 2014; Neraas & Spruell, 2001; Roberts et al., 2013), lower genetic diversity (Ardren & Bernall, 2017; Cena et al., 2006; Frankham, 1995), and even facilitate hybridization (Hasselman et al., 2014). One species affected by dams is the sauger (*Sander canadensis*), which has seen declines across its range associated with dam‐induced habitat fragmentation and habitat loss (McMahon & Gardner, 2001; Pegg et al., 1997). The reservoirs upstream of many large dams pose additional threats when sauger and walleye (*Sander vitreus*) occur in sympatry.

Walleye and sauger are sympatric across most of their respective ranges and generally spatially segregate from one another, with sauger choosing deeper water than walleye (Haxton, 2015). However, in highly altered environments like reservoirs, this spatial segregation diminishes and interspecific competition increases (Bellgraph et al., 2008; Butt et al., 2017; Gangl et al., 2000; Rawson & Scholl, 1978). The increased ecological overlap in reservoirs is often accompanied by hybridization between the two species; almost every documented instance of sauger × walleye hybridization has occurred within a reservoir or impounded section of river (Barton, 2011; Graham et al., 2020). Instances of hybridization in non‐impounded rivers have only been recorded in locations where sauger and walleye do not naturally overlap (Billington et al., 2006; Bingham et al., 2012; Koigi, 2004; McMahon & Gardner, 2001), which suggests that evolutionary history may also play a role in determining where hybridization will occur through the evolution of reproductive barriers in areas of native sympatry. Despite knowledge of where hybridization is likely to occur, the prevalence of sauger × walleye hybridization across their sympatric range is still unknown.

While some sauger × walleye hybrids can be identified phenotypically, misidentification of hybrids as parental species also occurs (Billington et al., 1997; White et al., 2005). A review of several studies that used morphological and molecular methods to detect hybrids found that morphological evidence always led to an underestimation of hybrid prevalence (Barton, 2011). Even when morphological characters are not used, previous genetic studies on sauger × walleye hybridization have mostly relied on only a few genetic markers, which makes identifying backcrossed or advanced generation hybrids difficult or impossible (McFarlane & Pemberton, 2019). Tools for molecular assessment of hybridization with enough resolution to reliably identify backcrossed individuals have only recently become widely available, and the advancement of DNA sequencing technologies over the past decade has allowed for detailed studies of hybridization in non‐model organisms. Hybridization has the potential to vary across geographic locations, through time, or in response to abiotic/biotic variables (Mandeville et al., 2017; Muhlfeld et al., 2014; Nolte et al., 2009; Sweigart et al., 2007), yet few studies have been explicitly designed to capture this potential variation. Studies of hybridization increasingly include sampling across multiple geographic locations, but similarly stratifying sampling through time is less common. If hybridization is found to vary spatially or temporally, it may be possible to develop conservation and management strategies that target crucial locations or time periods with management interventions (Mandeville et al., 2019). Therefore, achieving management goals for species threatened by hybridization may require reliable, high‐resolution estimates of hybrid status and a thorough understanding of the spatiotemporal variability of hybridization prevalence.

In Wyoming, the Bighorn and Wind Rivers system is an area of particular interest for sauger conservation. Despite longstanding concerns about hybridization and introgression of native sauger with walleye, previous studies have found very few hybrids (Bingham et al., 2012; Krueger et al., 1997). However, given evidence from other locations where walleye and sauger are sympatric (Billington et al., 1988, 1997; Graeb et al., 2010; Graham et al., 2020), there has been concern that previous sampling might have missed hybridization that was occurring, either due to a mistargeted sampling effort or due to limitations of older genetic methods. Additionally, sauger populations within this river system exhibit several different life histories and are fragmented by a series of dams in the upper Bighorn River and lower Wind River, including the 67 m tall Boysen Dam, none of which have fish passage alterations (Welker et al., 2001). In this study, we used spatially and temporally replicated sampling and genomic data to quantify hybridization between native sauger and introduced walleye and characterize differentiation between sauger populations and life histories. Additionally, we aimed to understand how population structure and hybridization prevalence were affected by dams within the river system.

## 2 MATERIALS AND METHODS

### 2.1 Study system

**FIGURE 1:**
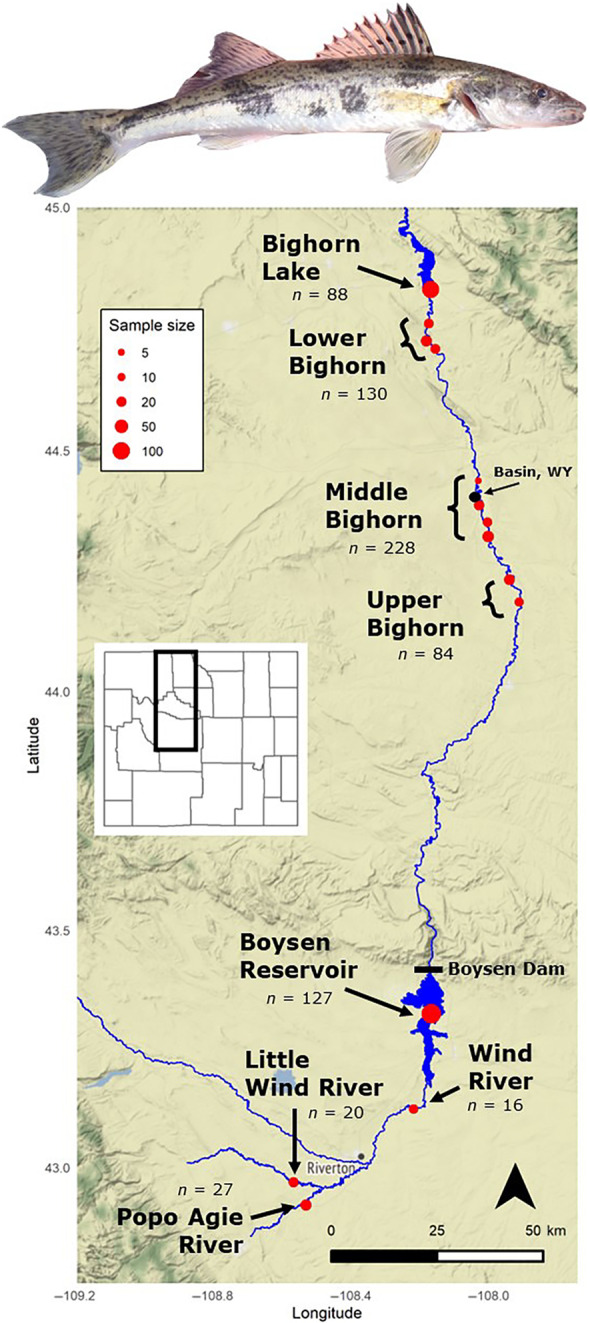
A photograph of a juvenile sauger above a map of the locations at which sauger and walleye were sampled. Red points indicate sampling locations, and the size of each point is relative to the number of samples collected at that location that were retained after sample processing, DNA sequencing, and subsequent filtering.

Within the Bighorn River (Figure 1), sauger are believed to have three distinct life histories. Some individual sauger spend the majority of the year in Bighorn Lake and migrate out to spawn in April and May and return shortly thereafter (Welker et al., 2001). Others migrate from Bighorn Lake in the spring to their spawning sites and return to the lake in the fall. A subset of sauger in the Bighorn River system are year round river‐residents and do not migrate to the lake during the year (Welker et al., 2001). Walleye are present throughout the river and in these observed spawning areas at much lower densities than the sauger (J.A.S., Wyoming Game and Fish Department, personal communication). Hybridization testing done on putative sauger parents for use in a 2014 spawning operation identified a single hybrid in the Bighorn River near Basin, Wyoming (Wyoming Game and Fish Department, 2015). Similar testing for spawning operations in 2015 and 2016 found no hybrid individuals (Bingham et al., 2018).

The Wind River sauger population has two distinct life histories. Reservoir‐resident fish spend their entire life, including spawning time, within the Boysen Reservoir (Lionberger, 2006). In contrast, river‐resident fish reside and spawn in the Popo Agie River and Little Wind River as adults, but most larvae drift down to Boysen Reservoir and the lower Wind River and reside there as juveniles, migrating back upstream as adults (Amadio et al., 2005, 2006; Kuhn et al., 2008; Lionberger, 2006). While no sauger–walleye hybrids had previously been observed within Boysen Reservoir, a coexisting walleye population is present (Bingham et al., 2012).

The Bighorn and Wind Rivers sauger populations are separated by Boysen Dam and a number of additional diversion dams along the mainstem Bighorn River that prevent movement between the populations. The connectivity of these populations was initially modified through the construction of the original Boysen Dam in 1908, which remained intact until a portion was blasted away in 1923 due to railroad flooding concerns. The current Boysen Dam was completed in 1952 just upstream from the original dam. There are believed to be no sauger or walleye, except presumably the rare fish moving downstream from Boysen Reservoir, between Boysen Reservoir and the most downstream of these diversion dams.

### 2.2 Sampling

Sampling occurred at four locations along the Bighorn River and on the Wind River system during fall 2017, spring 2018, and fall 2018 (Figure 1) to facilitate a thorough assessment of the genetic structure and extent of hybridization between sauger and walleye. We also included reference walleye samples from Fort Peck Hatchery in Montana in our genetic analyses.

Samples of both walleye and sauger were collected from the Bighorn River using boat electrofishing during three critical time periods including (1) before peak sauger spawning (early May), (2) peak sauger spawning (mid‐late May), and (3) when fish are on their home ranges (non‐spawning season, all other times of year). Genetics samples were collected from the Popo Agie, Wind, Bighorn, and Little Wind Rivers using boat electrofishing and in Boysen Reservoir using a combination of gill netting and shoreline electrofishing. Each fish was phenotypically identified as a sauger, walleye, or hybrid at the time of sampling by means of four characteristics: dorsal spots (walleye) or streaking (sauger), black spot at posterior of first dorsal fin (walleye) or no spot (sauger), white margin of caudal fin (sauger), or white spot at dorsal fin tip (walleye), and cheek scales present (sauger) or absent (walleye). Tissue samples (fin clips) were obtained from the first 100 individuals collected at each site and time period independent of phenotype. All fish were measured, weighed, photographed, and sexed (in accordance with approved animal handling protocols; IACUC permit number 20180917EM00328‐01 from the University of Wyoming). Fin clips were stored in 95% ethanol prior to DNA extraction. See Table 1 for the number of fish samples collected in each location on each day. Given the low number of sauger in the Wind River upstream of Boysen Reservoir, genetic samples that were opportunistically collected between 2012 and 2016 by the Wyoming Game and Fish Department were included in the genetic analysis.

**TABLE 1:** Table of number of samples collected by date and location.

Location	2012 through 2015	Jan. 2016	May 2016	Sept. 2016	May 2017	Sept. 2017	Oct. 2017	Jan. 2018	April 2018	May 2018	Sept. 2018	Oct. 2018	Nov. 2018
Bighorn Lake	0	0	0	0	0	0	0	0	0	0	0	0	91
Lower Bighorn	0	0	27	0	0	0	0	0	0	0	105	0	0
Middle Bighorn	0	0	60	0	30	0	0	0	0	0	128	0	12
Upper Bighorn	0	0	4	0	0	0	0	0	0	0	69	0	11
Boysen Reservoir	13	5	0	7	0	1	0	1	53	0	0	53	0
Little Wind River	0	0	0	0	0	0	20	0	0	0	0	0	0
Popo Agie River	0	0	0	0	0	0	27	0	0	0	0	0	0
Wind River	0	0	0	0	0	0	0	0	0	0	14	2	0

### 2.3 DNA extraction, library preparation, and Illumina sequencing

Genomic DNA was extracted from fin tissue using Qiagen DNeasy Blood and Tissue kits and a QIAcube robot according to the manufacturer's instructions (Qiagen, Inc.). Similar to other sequencing projects involving non‐model fish species (Mandeville et al., 2017; Underwood et al., 2016), we prepared reduced complexity genomic libraries for high throughput DNA sequencing using a genotyping‐by‐sequencing protocol (Parchman et al., 2012). DNA was initially fragmented using restriction enzymes EcoRI and MseI and fragments from each individual fish's DNA were ligated to a unique 8–10 base pair nucleotide barcode. Following the ligation of the identification barcodes, individual samples were multiplexed and amplified by PCR. Between 192 and 207 individuals were pooled per library and each library was sequenced on one Illumina HiSeq 2500 lane to produce approximately 1 billion 100 base pair sequence reads. Prior to sequencing, each library was size‐selected using BluePippin (Sage Science) to retain only fragments 250–350 base pairs in length. DNA sequencing of all four libraries (813 individual fish) was completed at the University of Texas Genome Sequencing and Analysis Facility, Austin, Texas.

### 2.4 Sequence assembly and estimation of genetic ancestry

DNA sequencing produced 1.118 billion reads. After parsing barcodes to assign each read to an individual fish, we retained 75% of the raw data. We then aligned reads from each individual fish to the yellow perch (*Perca flavescens*) genome (Feron, Zahm, et al., 2020) using the BWA‐MEM algorithm (Li, 2013), which resulted in alignment of 80% of reads to the reference genome. We then identified variable genetic sites using Samtools mpileup. We filtered the initial set of variants to include only SNPs (single nucleotide polymorphisms) with two alleles. We also applied filters using VCFtools to retain only loci with data in at least 50% of individuals and loci with a minor allele frequency of 0.03 or greater. Missing data were relatively evenly distributed within this dataset, as the average and median percent missing data per locus were 20.5% and 17.9%, respectively. For principal component analysis (PCA), we further filtered this dataset to include only loci with data in 75% or more of individuals. Missing data were relatively evenly distributed across loci in both datasets. Fourty‐five individuals missing data for 80% or more of these retained sites were removed from the dataset. Missing data were relatively evenly distributed across the remaining individuals (mean missingness 19.7%, median 17.9%). This and all following sequence processing was performed on the Univeristy of Wyoming's Teton Computing Cluster (Advanced Research Computing Center, 2018).

Initial analyses of genetic differentiation revealed genetic differentiation by sex, as expected if there are relatively large differentiated sex chromosome loci included in our sampling of the genome. We thus identified loci associated with sex‐linked genetic differentiation through a discriminant analysis of principal components (DAPC, Jombart, 2008) with sex as the grouping variable (Benestan et al., 2017). DAPC was performed using the R package adegenet (Jombart, 2008). We identified loci with DAPC loadings at or above the 0.99 quantile of all loadings using methods similar to those used in Junker et al. (2020). These sex‐linked loci were removed from downstream analyses of hybridization and of genetic differentiation within sauger populations. This DAPC approach was also used to identify the distribution of variants associated with additional unexplained population structure present in both Wind River and Bighorn River sauger populations.

To identify the ancestry of individual fish, in order to detect potential hybrids, we used entropy, a hierarchical Bayesian model (Gompert & Buerkle, 2013; Shastry et al., 2021). For each individual fish, we estimated q, proportion of ancestry, and *Q*, interspecific ancestry (proportion of genetic sites with ancestry from both parental species) for a *k* = 2 model assuming two genetic clusters (i.e., sauger and walleye). We used the bivariate relationship of *q* and *Q* to classify individuals into categories of hybrid crosses. Fish with a proportion of walleye ancestry (*q*) less than 0.1 were classified as sauger and those with proportions of walleye >0.9 were classified as pure walleye. All fish with proportions of walleye ancestry >0.1 and <0.9 were classified as hybrids. Hybrids were further broken down into F1, F2, and backcross (BC) classes using the combination of *q* and *Q* values. F1 hybrids were defined as fish with *q* between 0.4 and 0.6 and *Q* > 0.8. F2 hybrids also had *q* between 0.4 and 0.6, but had lower *Q* estimates, between 0.4 and 0.6. BC sauger were defined as *Q* − 2*q* between −0.1 and 0.1; BC walleye were defined by *Q* + 2*q* between 1.9 and 2.1. These cut‐offs were selected to be similar to previous work (Mandeville et al., 2019; Rosenthal et al., 2022) and derived from work on the entropy model evaluating realistic expectations for these values (e.g., Lindtke et al., 2014). These classes are broad by necessity due to the effects of genetic variation in parental populations when sampling thousands of loci. Variation within each parental species can cause truly unadmixed individuals to score as >0.00 or <1.00. This can then propagate to deviations in hybrid individual admixture coefficients (e.g., F1 hybrids with *𝑞* ≠ 0.5).

To assess genetic differentiation within sauger populations, we next excluded all walleye and hybrids from the dataset and used a more stringent missing data filter (maximum allowed missing data per locus of 0.25). We assessed population structure between Wind River Basin and Bighorn Basin sauger populations in several ways. First, we used PCA to explore patterns of spatial genetic structure and divergence between life history strategies in the data. We used R to calculate a genotype covariance matrix among all individuals, then completed a PCA of that genotype covariance matrix using the prcomp function in R (similar to methods used in Mandeville et al., 2017). Principal components one through four were examined for each analysis. We also performed PCA on subsets of sauger individuals based on their sampling location and sampling date, in effort to detect low‐level differentiation that might not be detected in an analysis with all individuals. We also examined the effects of library preparation on PCA results by looking for correspondence between sequencing library and flow cell lane and PCA cluster assignment.

We calculated pairwise *F*_ST_, a measure of genetic distance, between populations from all sampling sites, and between putative life history types, using the Reich‐Patterson *F*_ST_ estimator (Reich et al., 2009). We used custom R scripts, based on those used in the R package dartR (Gruber et al., 2018), to test for a significant difference of *F*_ST_ values from zero via bootstrapping.

We looked at evidence for continuous population genetic structure with distance by examining the relationship between genetic divergence and river distance between populations (isolation by distance), testing this relationship using Mantel tests to account for spatial autocorrelation in the data. Genetic divergence was estimated by *F*_ST_/(1−*F*_ST_) (Rousset, 1997), where *F*_ST_ was calculated in the same manner as before. River distance was calculated using the R package riverdist (Tyers, 2017). Analyses of isolation‐by‐distance (IBD) were done to assess divergence along the Bighorn River and throughout the Wind/Bighorn River system.

We calculated metrics of genetic diversity, Watterson's estimator (*𝜃*_*𝑊*_) and Tajima's *𝜋*, using ANGSD (Korneliussen et al., 2014) to compare the relative genetic diversity of sauger populations above and below Boysen Dam. Observed heterozygosity was also calculated for each individual using ANGSD; individual values were averaged to create a population‐wide average observed heterozygosity for each population.

### 2.5 Demographic modeling

To better understand the potential effects of Boysen Dam on sauger population structure, we constructed demographic models in dadi (Gutenkunst et al., 2010) to estimate the divergence time between sauger upstream and downstream of Boysen Dam. We operated under the assumption that if the divergence was not caused by Boysen Dam, we should expect to only see divergence time estimates much older or much younger than the dam (109 years before the samples were collected). We constructed a total of three different demographic models. The most complex model featured a population splitting into two populations, where the resulting two populations had a total size smaller than or identical to (but not larger than) the starting population size. Populations then evolved separately with asymmetric, bidirectional migration. The other model was nested within the more complex model: no migration was allowed after divergence. Because this models was nested within the most complex model, we performed a log‐likelihood ratio test (α = 0.05) to measure whether the added parameters sufficiently increased our model fit.

The genomic data used for the demographic model fitting were slightly different from that used in other analyses: we did not filter by minor allele frequency or remove sex‐associated loci. We also retained SNPs with as much as 70% missing data. We used the easySFS tool (http://www.github.com/isaacovercast/easySFS) to generate the input file for dadi and to examine the effects of changing site frequency spectrum sample sizes on the number of segregating sites between our populations. Because there is no mutation rate estimate for sauger or other closely related species, we took the approach used by Zhao et al. (2020) and included several mutation rates from different taxa with varying mutation rates, including humans (2.5 × 10^−8^; Nachman & Crowell, 2000), cichlids (3.5 × 10^−9^; Malinsky et al., 2018), and Atlantic herring (*Clupea harengus*; 2 × 10^−9^; Feng et al., 2017). We also included a recent estimate of the average fish mutation rate (5.97 × 10^−9^; Bergeron et al., 2023). Converting dadi parameter estimates to real‐time also requires an estimate of the total sequence length used to generate the site frequency spectrum. We estimated this length by retaining all sites (variant and invariant, no filter for missing data; “total sites file”). Because we filtered the SNP VCF file for missing data prior to generating the site frequency spectrum, our total number of sites should exclude sites that would be lost with missing data filtering. To estimate this, we multiplied the number of sites in the total sites file by the proportion of SNPs retained after filtering the SNP‐only VCF file (number of SNPs post‐filtering divided by the number of SNPs pre‐filtering). This resulted in a value of 25,442,985 base pairs. We assumed a generation time of 3 years to convert divergence time estimates from generations to years. To estimate the parameters within each model, we used the LN_COBYLA algorithm from the nlopt library (Johnson & Schueller, 2021). We calculated the uncertainty of our parameter estimates bootstrapping our data 100 times over one megabase fragments and subsequently using the Godambe GIM approach implemented in dadi. We examined the residuals for each model to ensure that our models were fitting the data reasonably well. We fit each model and performed log‐likelihood ratio tests 26 times to ensure that parameter estimates converged on the global optimum rather than local optima.

## 3 RESULTS

**FIGURE 2:**
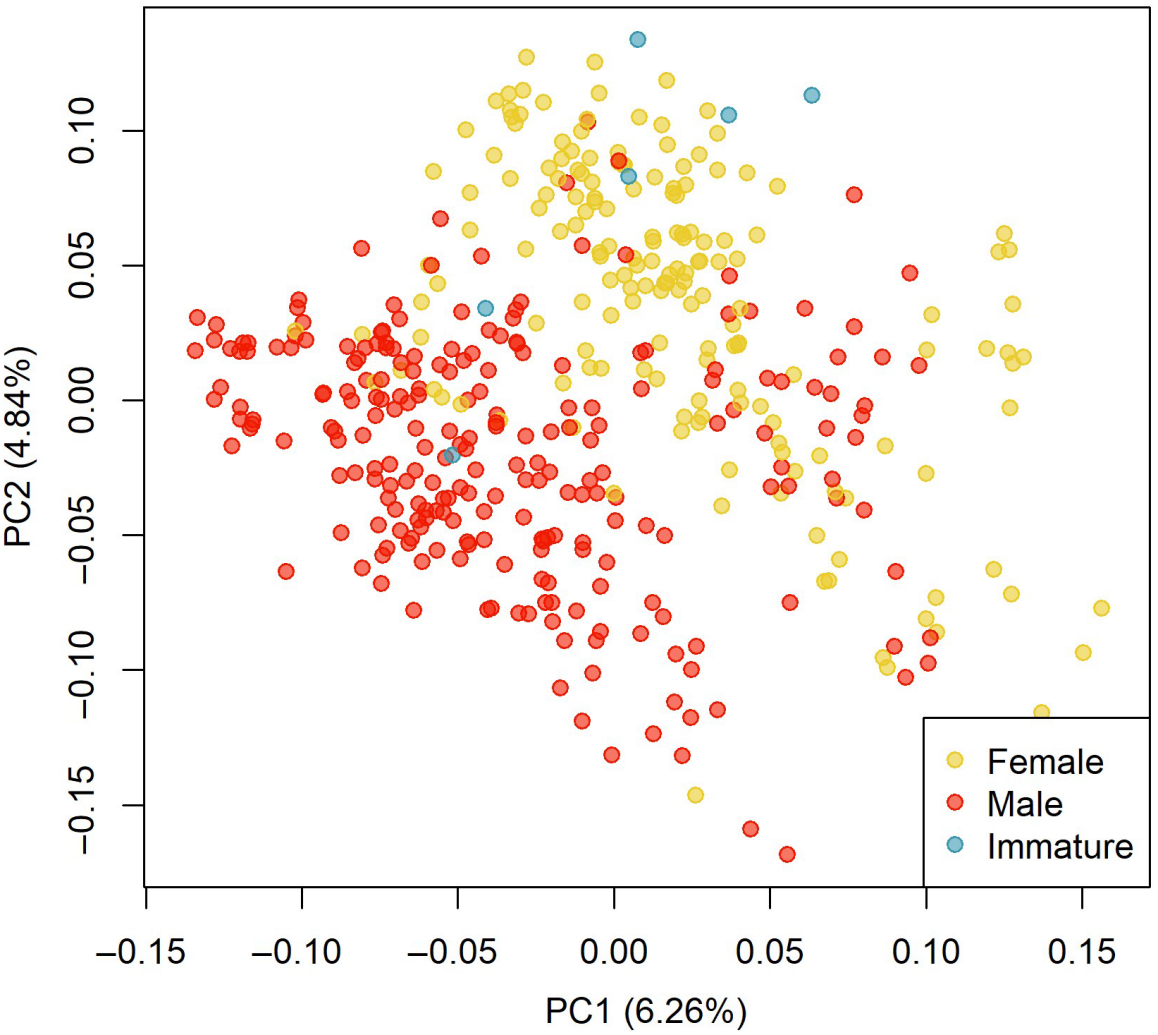
Principal components analysis (PCA) of 6627 genomic loci for the full sauger dataset revealed differentiation associated with sex of the sauger individuals. Each point represents one individual with recorded sex indicated by color. The percent of total variance explained by each principal component is indicated on the *x* and *y*‐axis labels. The loci contributing to the divergence between sexes were removed for further analyses of sauger population structure.

**FIGURE 3:**
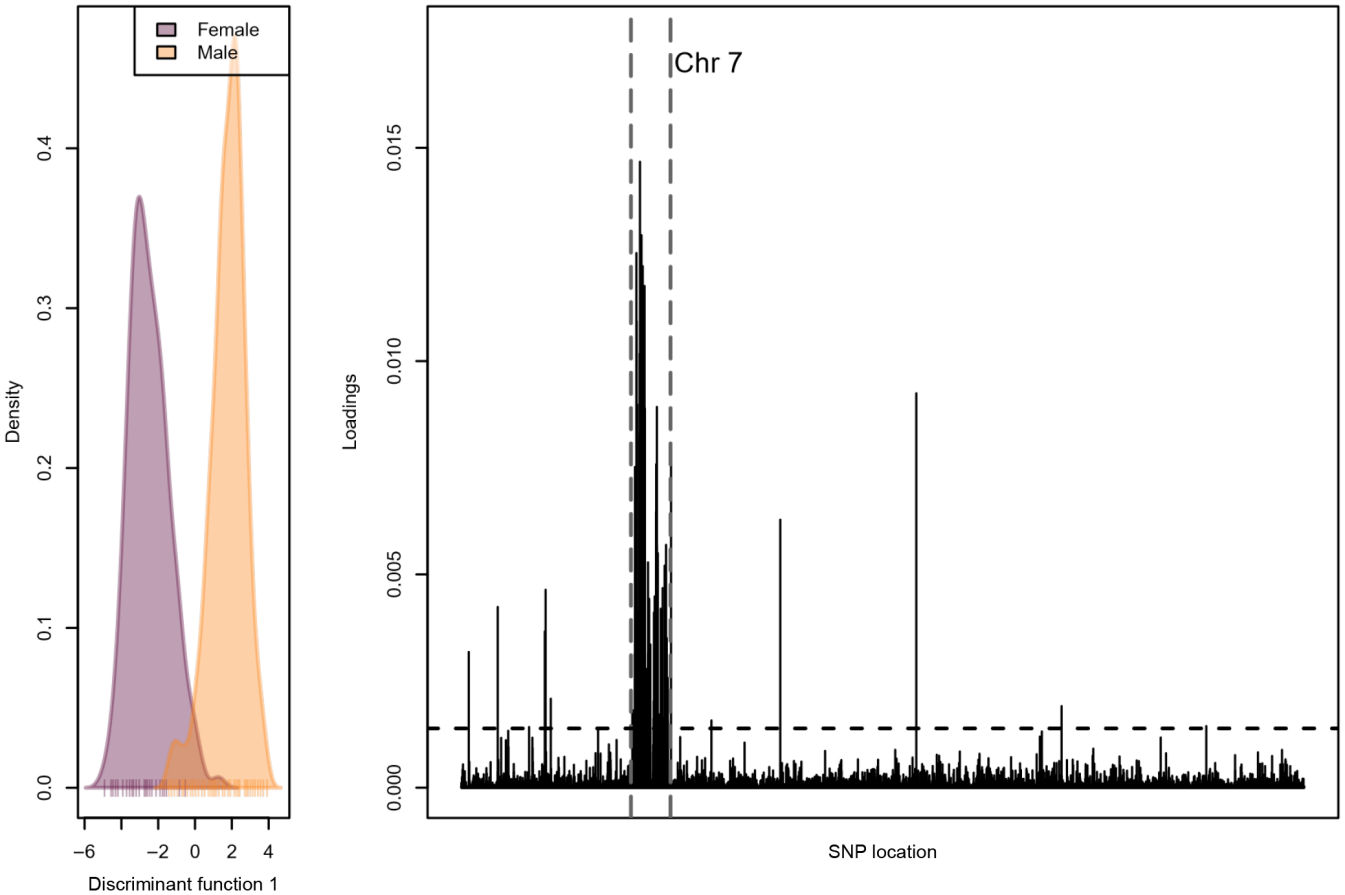
Discriminant analysis of principal components results for all sauger either identified as male or female. The left window shows the distribution of individuals along the first discriminant function (colored by sex), and the right window shows the discriminant function loading for each SNP across the genome. The yellow perch chromosome 7 is highlighted between vertical dashed gray lines, and the horizontal dashed black line shows the significance threshold obtained from a DAPC randomization procedure.

Before filtering, we identified 2,255,833 SNPs. After filtering for minor allele frequency (minimum 0.03) and missing data (maximum 50% missing per locus), we retained 17,271 SNPs. This same filtering scheme resulted in only 6536 SNPs when only unadmixed sauger individuals were included. The more heavily filtered dataset used for principal components analysis (maximum 25% missing data per locus) contained 4152 SNPs. Filtering individuals that sequenced poorly (70% missing data or more) brought the total number of samples down from 813 to 768, of which 720 were wild individuals (as opposed to hatchery reference walleye). Initial examination of the genomic data through principal components analyses revealed genetic differentiation linked to sex rather than population of origin (Figure 2), leading us to remove sex‐associated loci from subsequent analyses of hybridization and population genetic structure. A total of 91 loci were removed, of which 79 were found on chromosome 7 of the yellow perch reference genome (Figure 3).

### 3.1 Hybridization with introduced walleye

**FIGURE 4:**
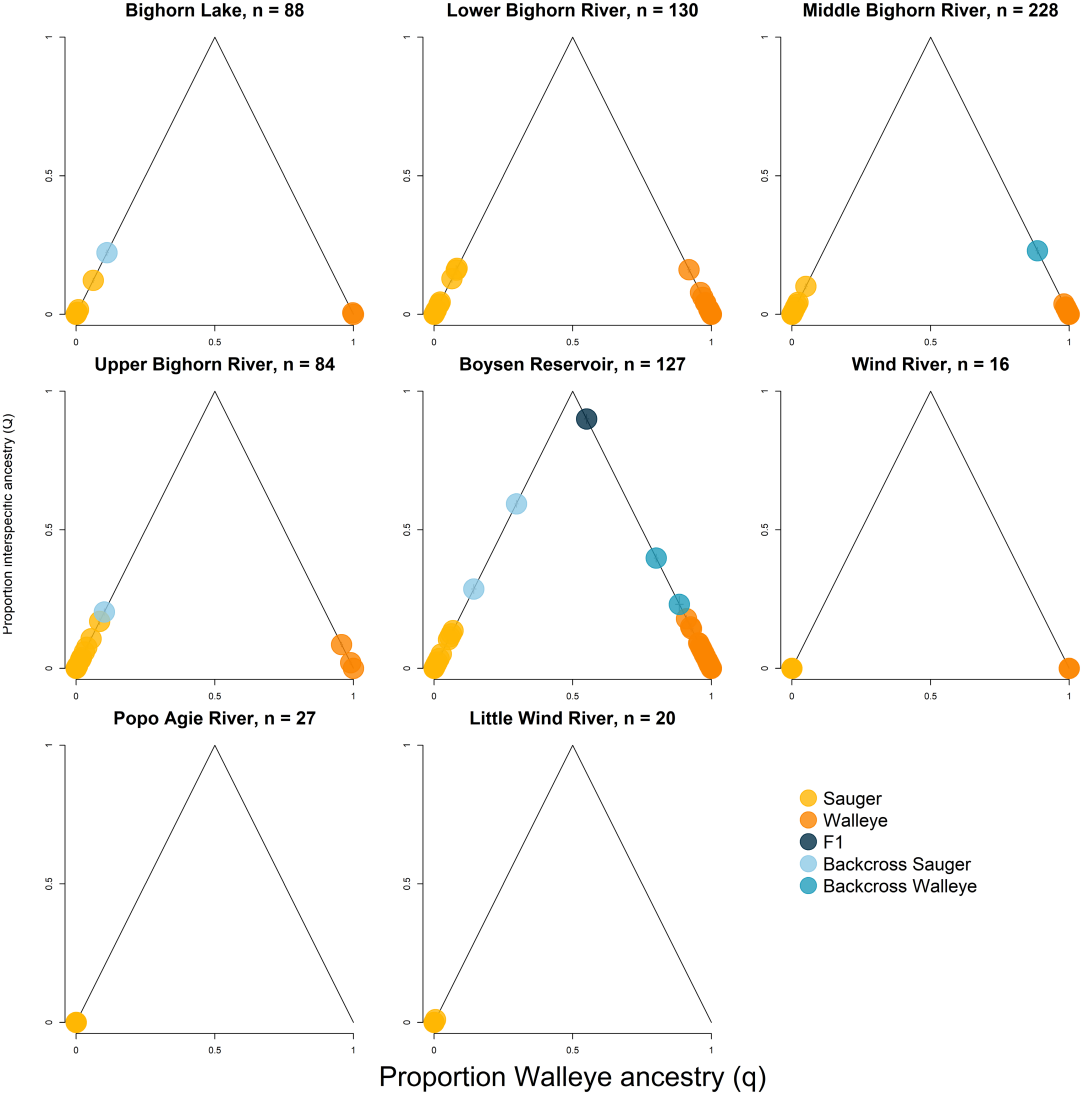
Plots of individual ancestry, as measured by entropy, for each sampling region. The *x*‐axis for each plot represents the proportion of an individual's genomic data that can be inferred as being of walleye origin. The *y*‐axis of each plot represents the proportion of loci in each individual that has ancestry from both sauger and walleye, which can be used to infer the recency of breeding between pure sauger and pure walleye individuals. Each individual is represented by a point colored by its ancestry class.

**FIGURE 5:**
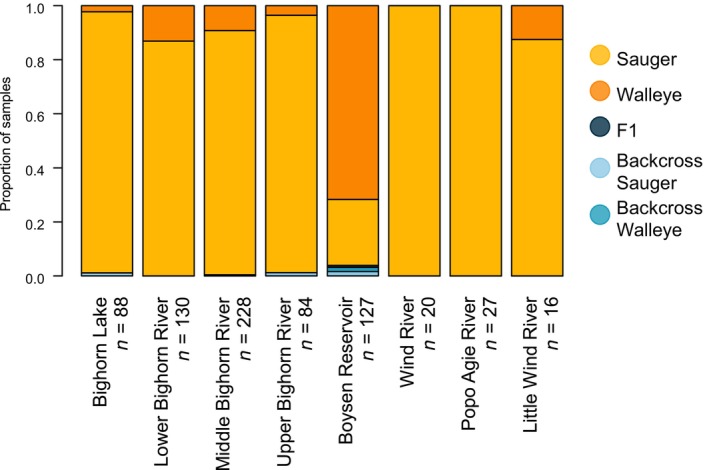
A bar plot showing the proportion of total samples per species, as identified by analysis of genetic data with entropy, at each location.

Of 720 sauger/walleye individuals from the Bighorn and Wind Rivers, eight were genotypically identified as hybrids by analyses using entropy (Figure 4). Of these eight, five were sampled in Boysen Reservoir (Figure 4). Boysen Reservoir was also the only location where an F1 hybrid and two additional intermediate hybrids were detected and is the location with the highest proportion of walleye samples (Figure 5). None of the detected hybrids were the product of a hybrid × hybrid mating, but instead appear to result from backcrossing to parental species. Three potentially hybrid individuals were sampled from the Bighorn River, but each had only a small proportion of walleye ancestry (0.11–0.15), as would be typical of advanced backcrosses, which would be the product of hybrids mating with individuals from parental species across several generations. Only one genetically identified hybrid was sampled in spawning season, in the middle Bighorn River. The other two potential hybrids from the Bighorn River system were sampled in Bighorn Lake and the Upper Bighorn River in November 2018 (Table 2). All individuals genetically identified as hybrids were not phenotypically identified as such. Of the eight identified hybrid individuals, five were phenotypically identified as walleye and three were phenotypically identified as sauger (Table 2). Those identified as sauger had low proportions of walleye ancestry (0.101–0.143).

**TABLE 2:** Additional information for each fish identified as a hybrid.

Sample ID	Location	Temporal event	Phenotypic ID	Date sampled	Sex	Maturity	*q*	*Q*
EGM18_2385	Boysen Reservoir		Sauger	10 Sept. 2015	F	Mature	0.143	0.286
EGM18_2522	Boysen Reservoir		Walleye	11 Sept. 2018	F	Mature	0.884	0.231
EGM18_2529	Boysen Reservoir		Walleye	11 Sept. 2018	F	Mature	0.550	0.899
EGM18_2550	Boysen Reservoir		Walleye	12 Sept. 2018	F	Immature	0.801	0.398
EGM18_2562	Boysen Reservoir		Walleye	12 Sept. 2018	M	Mature	0.297	0.593
EGM18_0187	Middle Bighorn	Spawn	Walleye	18 May 2018	M	Immature	0.885	0.230
EGM18_2663	Bighorn Lake		Sauger	6 Nov. 2018			0.111	0.222
EGM18_2684	Upper Bighorn	Nonspawning	Sauger	8 Nov. 2018			0.102	0.203

### 3.2 Population structure in sauger populations

**FIGURE 6:**
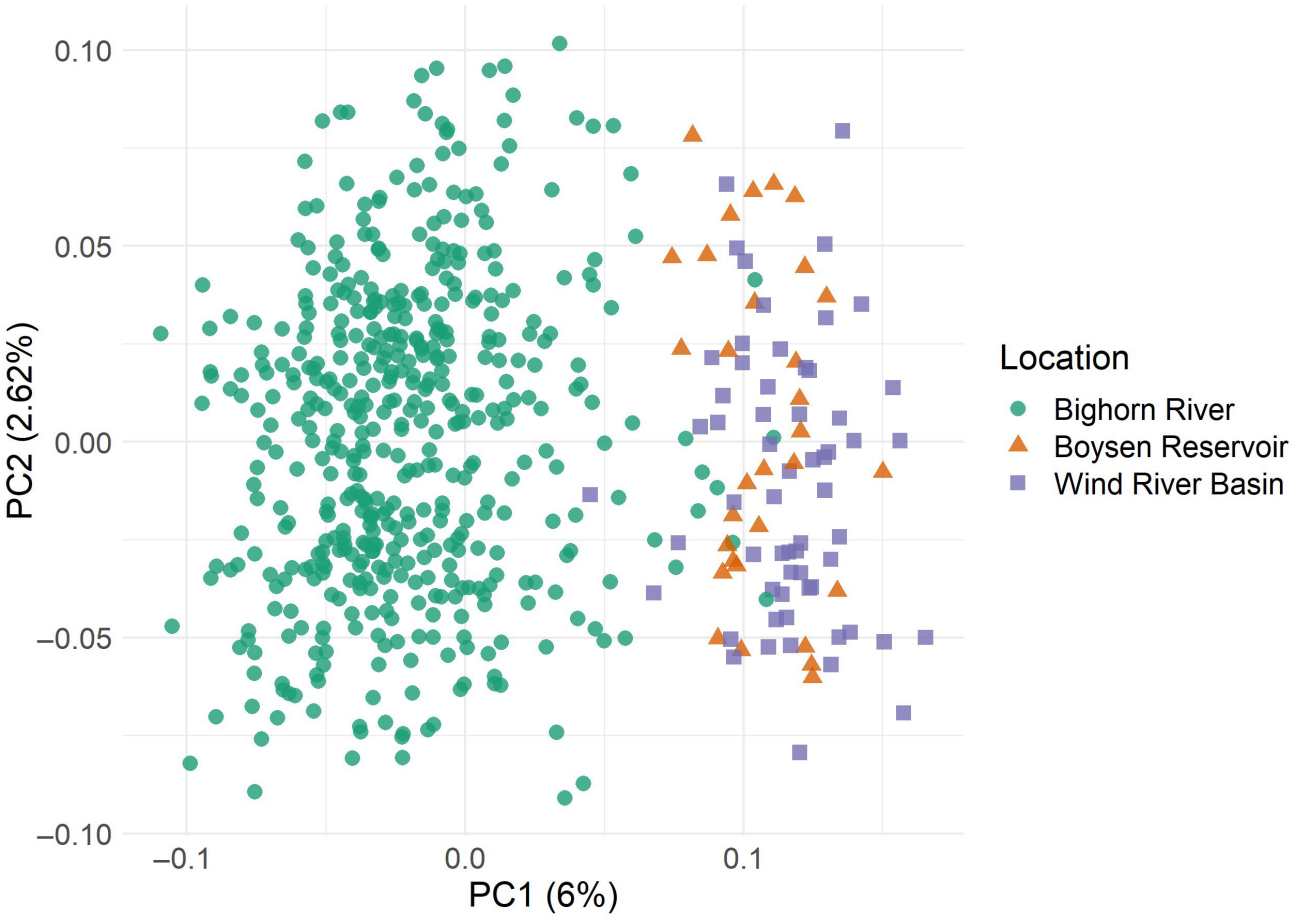
Principal component analysis (PCA) of 4179 genomic loci in sauger individuals after the removal of sex‐associated loci reveals population structure differentiating Bighorn River and Boysen Reservoir/Wind River sauger populations. Each point represents one individual, and each point is colored by its origin. The Bighorn River group includes individuals sampled in the lower, middle, and upper Bighorn regions, as well as Bighorn Lake. The Wind River Basin group includes individuals collected in the Wind River, Little Wind River, and Popo Agie River. The percent of total variance explained by each principal component is indicated on the *x* and *y*‐axis labels.

**FIGURE 7:**
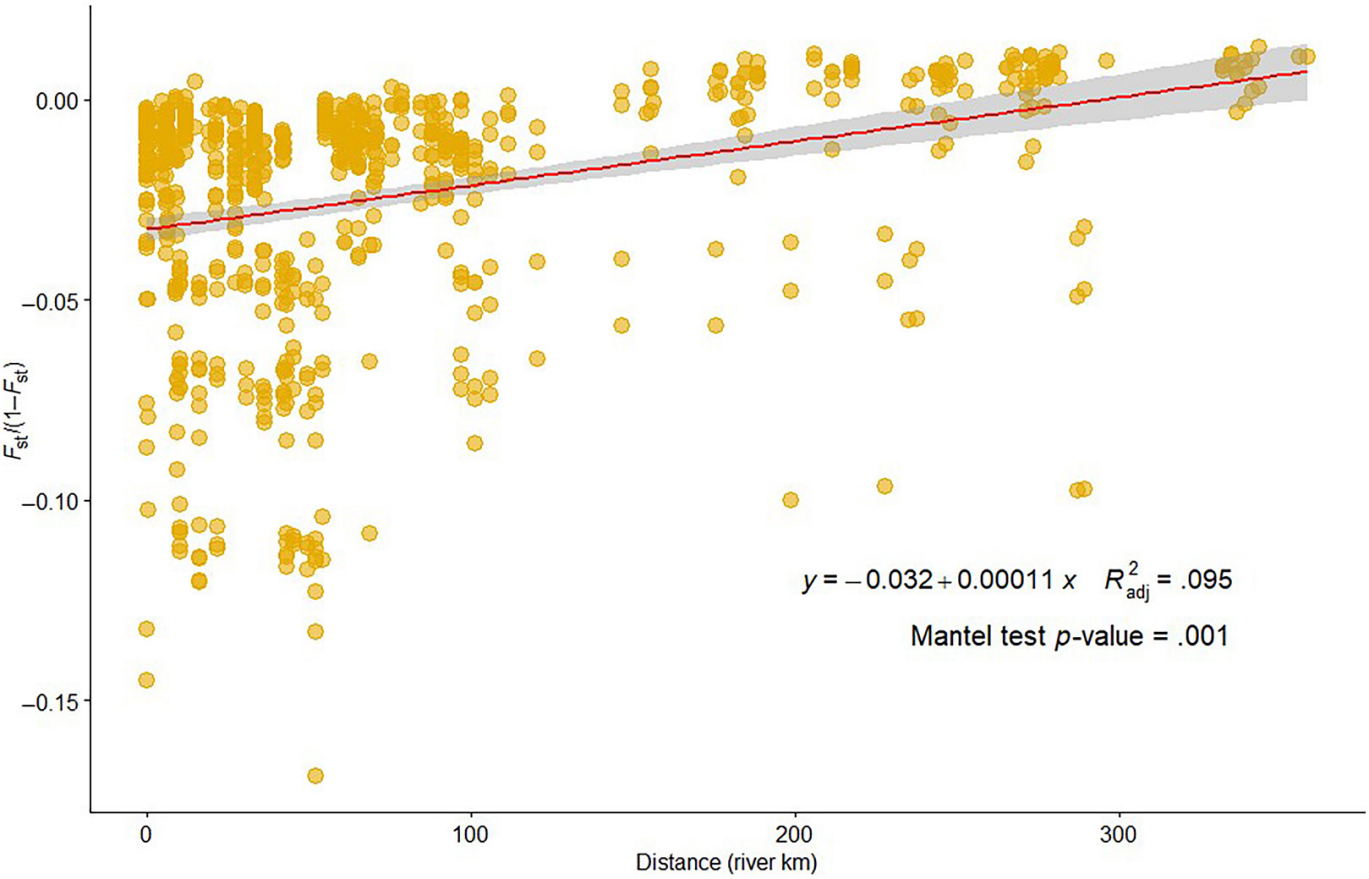
Assessment of isolation‐by‐distance for sauger across all sampling locations. Each point represents a comparison between two sampling locations, with the river distance between them on the *x*‐axis and their divergence (as measured by *F*_st_) on the *y*‐axis. The line shows a linear regression of *F*_st_ as a function of distance. A mantel test to account for spatial autocorrelation of sampling points reveals that this relationship is significant (*p* = .001).

The PCA highlighted genetic differentiation between sauger living in the Bighorn River and in Boysen Reservoir and the Wind River Basin (Figure 6). However, the overlap of the two clusters indicates that the divergence between these populations is not strong. The estimated *F*_𝑆𝑇_ between sauger in the Wind River and Bighorn Basins was only 0.0110 (95% credible interval 0.0110, 0.0117). Relatively weak divergence between populations, in this case, suggests a recent populations split. Isolation‐by‐distance analysis showed a significant effect of distance on genetic divergence between populations when sauger from all locations were included (Mantel test, *p*‐value = .001; Figure 7), but did not show a significant relationship within spawning fish in the Bighorn River sauger (Mantel test, *p*‐value = .917).

Estimates of genetic diversity differed greatly between populations above and below Boysen Dam: *𝜃*_*𝑊*_ and *𝜋* were two orders of magnitude lower in sauger upstream of Boysen Dam (*𝜃*_*𝑊*_ = 0.000396, *𝜋* = 0.000673) than in sauger downstream of Boysen Dam (*𝜃*_*𝑊*_ = 0.022135, *𝜋* = 0.003775; Table 3). Expected heterozygosity showed no such pattern and was similar for sauger upstream of Boysen Dam (0.2718) and below Boysen Dam (0.2743).

**TABLE 3:** Diversity metrics for unadmixed sauger populations upstream and downstream of Boysen Dam.

Location	𝝅	𝜽_𝑾_	Observed heterozygosity
Wind River (upstream of Boysen Dam)	0.000673	0.000396	0.000328
Bighorn River (downstream of Boysen Dam)	0.003775	0.022135	0.000328

Our analyses detected no clear genomic differentiation between sauger exhibiting different life history strategies in the Bighorn River. PCA of Bighorn River sauger color‐coded by spawning location did not reveal any clear clustering. Estimates of *F*_𝑆𝑇_ between groups of sauger collected at different locations during the spawning period were not statistically different from zero, with the exception of one comparison between sauger collected at the middle Bighorn location between river miles 46 and 55 and those collected at the lower Bighorn location between river miles 9 and 11 (*F*_𝑆𝑇_ estimate of 0.00319, 95% confidence interval of 0.00108 to 0.00512). Likewise, there is no clear differentiation present between lower, middle, and upper Bighorn sauger during the pre‐spawning period, or during the non‐spawning season. When examined by collection location, there is no structure evident between pre‐spawn, spawning, and non‐spawning fish. There appears to be a small degree of clustering of spawning sauger sampled in the same year in the Bighorn River. A similar pattern was observed in sauger collected in Boysen Reservoir and the Wind River Basin; no clear differentiation was detected between sauger collected in Boysen Reservoir, the Wind River, the Popo Agie River, or the Little Wind River.

Weak genetic differentiation was also observed on PC3 in both the Bighorn and Wind Rivers populations. The variants contributing to this differentiation were found across most of the genome. The magnitude of this divergence is slightly lower than that found above and below Boysen Dam (*F*_𝑆𝑇_ estimate of 0.00815, 95% confidence interval of 0.011 to 0.006).

### 3.3 Demographic modeling

Filtering our genomic data for demographic modeling purposes retained 275,782 sites. We projected down to 290 samples for the Bighorn River (downstream of Boysen Dam) and 54 samples for the Wind River (upstream of Boysen Dam). This level of down‐projection was necessary to prevent the missing data in our SNP dataset from affecting our site frequency spectrum.

Of the 26 independent rounds of model fitting, the log‐likelihood ratio tests supported the more complex model over the simple model (divergence with no gene flow) in only two rounds. However, these two rounds were among the six total rounds where the log‐likelihood of the simple model was much lower than the other rounds, suggesting that the optimizer found a local optimum rather than the global optimum. For the other 20 rounds that did appear to find the global optimum for the simple model, a complex model was never supported by the log‐likelihood ratio test (*p*‐values > .3) and model residuals appeared appropriately distributed. Given these results, we concluded that the simple model (divergence with no migration) was the most appropriate one to consider moving forward.

**FIGURE 8:**
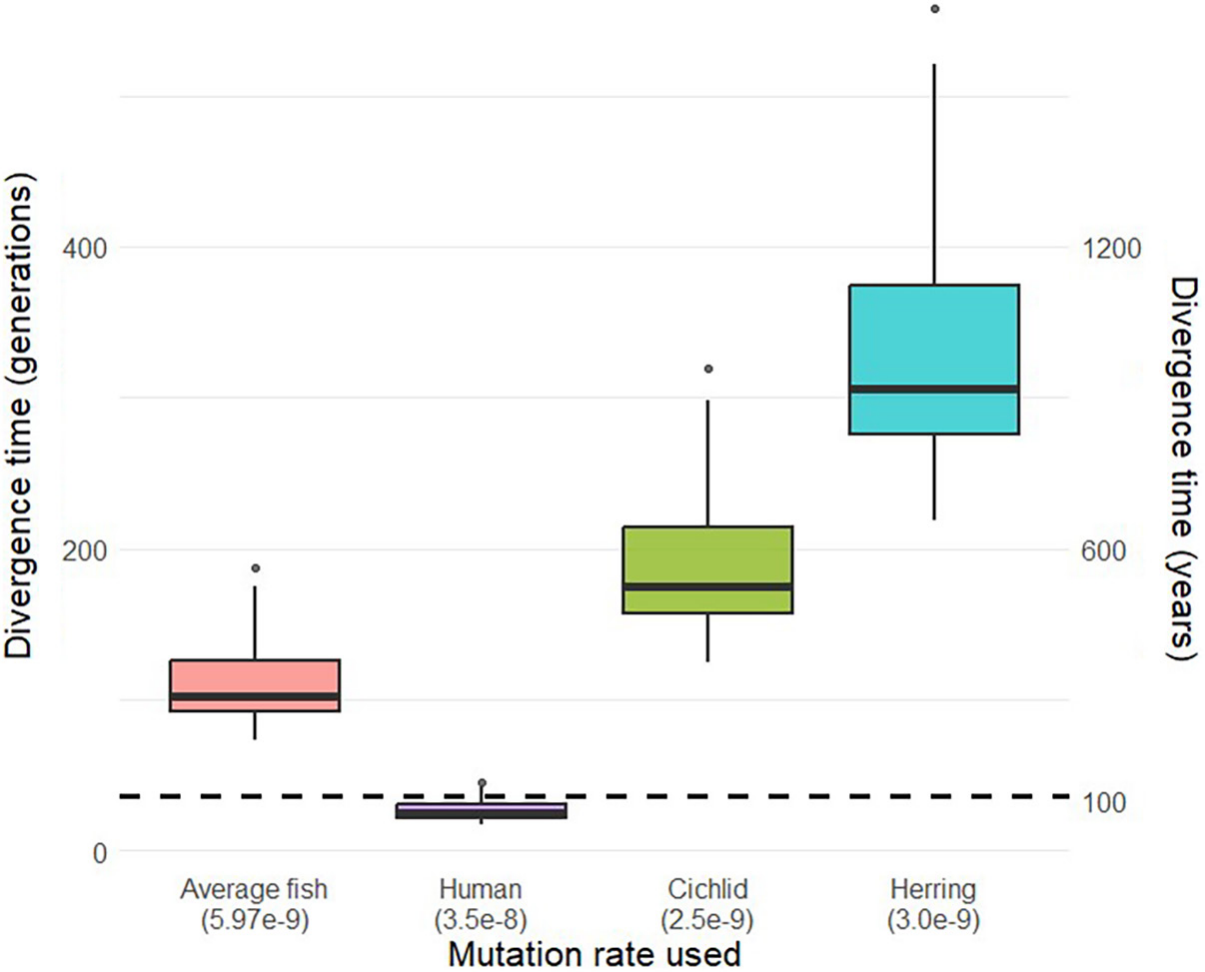
Divergence time estimates from 20 demographic models of sauger populations upstream and downstream of Boysen Dam. Each box in the plot shows the results for all 20 models, but with a different mutation rate used to scale parameter estimates to sauger generations. The second *y*‐axis shows those same estimates scaled to years using a generation time of 3 years, and the dashed horizontal line shows the time of Boysen Dam's original installation.

Divergence time estimates varied substantially depending on the mutation rate used, but fell between 17.5 and 557.8 generations (52.5 and 1673 years, respectively; Figure 8). Parameter estimate uncertainty was very low across all model fitting rounds (average 95% confidence interval width of 2.41 × 10^−7^, or 0.003 generations given the average fish estimated mutation rate), though the difference in estimates between rounds was higher than this uncertainty.

## 4 DISCUSSION

In this study, we assessed sauger population structure and hybridization rates with introduced walleye, focusing on the potential for dams to impact spatial connectivity. Additionally, our rigorous temporal and spatial sampling allowed us to examine potential variation in hybridization associated with geographic locations and sauger life histories. Previous studies have detected very little hybridization in the Bighorn River system despite sympatry of sauger and walleye (Bingham et al., 2012; Krueger et al., 1997; Wyoming Game and Fish Department, 2015), but given that these species are known to hybridize elsewhere where they are sympatric (Billington et al., 1997; Bingham et al., 2012; Graeb et al., 2010), concerns have persisted about the potential threat that introgressive hybridization might pose to native sauger populations. The Bighorn and Wind Rivers drainage system is considered the last stronghold for native sauger in Wyoming (Bingham et al., 2018; Welker et al., 2001), so accurately quantifying the threat of hybridization with walleye and fully describing population genetic structure in native sauger is essential for the conservation and effective management of native sauger populations. We find evidence for minimal hybridization between sauger and walleye in this region. The few hybrid individuals that we identify seem to be found disproportionately in reservoir environments with higher walleye abundance. Additionally, we find evidence for intraspecific spatial differentiation in sauger associated with dams that impact fish movement. We discuss these and other results below.

### 4.1 Hybridization is present, but infrequent

Our results suggest that the Bighorn and Wind Rivers system has low levels of hybridization between sauger and walleye. Given sympatry between these species, including extensive overlap in spawning time and locations in the Bighorn River, it is noteable that hybridization is not more extensive. The low levels of hybridization that we detect are in agreement with previous studies (Bingham et al., 2012; Krueger et al., 1997). Only eight individuals (about 1% of sampled individuals) across the Bighorn and Wind Rivers system were designated as hybrids by the criteria of our analyses (Figure 4). A caveat to these results is that all three of the Bighorn River hybrid individuals had proportions of walleye ancestry within 2% of our boundary between hybrid and unadmixed sauger/walleye ancestry. The precision of these ancestry estimates is exceedingly high (the 95% credible interval widths for these estimates are less than 0.02), but our cutoff for labeling an individual as a hybrid is necessarily subjective, and it is possible that individuals with ancestry values just exceeding the thresholds might actually be unadmixed sauger or walleye. We believe that these thresholds for ancestry values (*q*/*Q*) are reasonable (Mandeville et al., 2019), but estimates of *q*/*Q* are sensitive to the amount of variation in reference parental species (Lindtke et al., 2014; Mandeville et al., 2017). Our reference parental samples included sauger and walleye from multiple locations, providing realistic variation in our reference panel.

Over half (5/8) of the identified hybrids were found in Boysen Reservoir. Three of the five Boysen Reservoir hybrids have greater than 10% of ancestry estimated in each of the two genetic clusters. These three individuals can be unquestionably identified as hybrids. These patterns suggests spatial variation in hybridization dynamics, with more hybridization occurring in Boysen Reservoir than anywhere else in the study area. Boysen Reservoir also had the highest walleye abundance within the study area, and this may explain variation in hybrid prevalence with the concentration of detected hybrids in this location (Figure 5).

There is little evidence to support the idea that certain life histories of sauger and walleye in the Bighorn River might be more susceptible to hybridization than others. Indeed, there is little evidence of hybridization in the Bighorn sauger populations at all (Figure 4), and as discussed below, genetic structure within the Bighorn river suggests frequent mixing among life histories. It is possible the hybrid individuals detected downstream of Boysen Dam were produced in Boysen Reservoir and passed through the dam during spillway releases; one such event did occur in 2017, before sampling for this project began (J.A.S., Wyoming Game and Fish Department, personal communication).

### 4.2 Intraspecific variation in sauger

A secondary goal of this study was to describe population genetic structure of sauger within this system to facilitate their management. We found relatively little spatial genetic structure within sauger in this system, but we did find detectable genetic differentiation separating populations above and below Boysen Dam (Figures 6 and 7). The degree of genetic differentiation is low and likely reflects recent divergence; low levels of divergence are consistent with previous studies that also imply differentiation corresponding to movement impacted by Boysen Dam (Bingham et al., 2012). The first dam at the mouth of the Wind River was built in 1908, although connectivity was likely briefly restored between when the original dam was removed in 1948 and when the new dam became operational in 1951 (Simon, 1951), and another partial restoration of connectivity may have occurred in 1923 when the dam was modified to alleviate railroad flooding. This history means that genetic differentiation reflects no more than 109 years of population subdivision since the original dam was built, or 55 to 22 generations. Anecdotal descriptions imply extensive movement of sauger throughout the Wind and Bighorn Rivers before the dam's installation (Simon, 1951). Although it is possible that the genomic differentiation between sauger upstream and downstream of Boysen Dam is related to ecological differences between the two populations, given that this divergence coincides spatially with Boysen Dam we believe that the differentiation we are seeing is more likely due to genetic drift within these finite populations separated by a barrier. Our demographic modeling results also support dam‐driven divergence of these populations, as the time estimates for average fish mutation rate are quite close to the time of Boysen Dam's original construction and the time estimates assuming a human mutation rate do overlap with the dam's original construction (Figure 8).

Genetic diversity estimates for sauger also differ substantially between populations upstream and downstream of Boysen Dam. Estimates of genetic diversity as measured by Watterson's estimator and nucleotide diversity (*𝜋*) were approximately two orders of magnitude lower above Boysen Dam (Wind River) than below it (Bighorn River and Bighorn Lake; Table 3). This pattern is consistent with a smaller effective population size upstream of Boysen Dam. Genetic diversity upstream of Boysen Dam is likely impacted by the major population declines of sauger in the Wind River system in the early 2000s; estimates of population size declined 73% from 2002 to 2010 (Gerrity & Smith, 2013). Alternatively, low genetic diversity within this population may have led to the observed declines. Low genetic diversity may negatively impact fitness (Reed & Frankham, 2003), but this is not always the case – the immediate effects of genetic diversity on fitness are still poorly understood (Leffler et al., 2012), and may be context‐dependent (Kardos et al., 2021; Teixeira & Huber, 2021). We observed no difference in average observed heterozygosity between populations upstream and downstream of Boysen Dam, though both estimates were extremely low (approximately 0.0003; Table 3). Bingham et al. (2012) also did not identify the difference in genetic diversity (as measured by expected heterozygosity) between Wind River and Bighorn River sauger, but saw much higher levels of expected heterozygosity than we see in our observed heterozygosity measures. This discrepancy can be at least partially attributed to the very different set of genetic markers used for our study (many SNPs) than for previous work (microsatellites). The pattern we have observed in this system is consistent with other studies suggesting low genetic divergence across similar spatial scales in sauger (Bingham et al., 2012) and other highly mobile fish species (Gouskov & Vorburger, 2016; Wolter et al., 2003).

Notably, there does not appear to be substantial differentiation within either the Bighorn River, where sauger are known to have several distinct life histories (Welker et al., 2001), or within the Wind River, where two different life histories are known to exist (Amadio et al., 2005, 2006; Kuhn et al., 2008; Lionberger, 2006). While these different life histories are an ecological reality (Amadio et al., 2005, 2006; Kuhn et al., 2008; Lionberger, 2006; Welker et al., 2001), according to our genetic data, this life history variation does not correspond to genetic structure, suggesting that either individuals switch spawning locations across years, or that spawning location is not heritable across generations.

In removing sex‐associated loci from the dataset prior to analyses of hybridization and population genetic structure, we learned that all sex‐linked loci in sauger are located on chromosome 7 of the reference yellow perch genome. Walleye showed no such pattern: sexes were not well‐differentiated by DAPC and loci driving any divergence were found across the yellow perch reference genome. A lack of sex‐associated loci in walleye was also found by Feron, Pan, et al. (2020) using the RADSex pipeline. Interestingly, chromosome 7 is not involved in sex determination in yellow perch (Feron, Zahm, et al., 2020). In general, fishes are known to have extremely variable sex determination mechanisms (Bachtrog et al., 2014), and differences among closely related species are common. Walleye and sauger are sister taxa estimated to have diverged during the Middle Miocene (approximately 15.4 million years ago; Haponski & Stepien, 2013). Our results thus suggest substantial differences in the genomic location of sex determination loci in sauger and walleye, if not differences in the mechanism of sex determination itself.

Aside from sex‐related differentiation, the most substantial genetic differentiation between sauger populations in the Wind and Bighorn drainages was associated with dams (Figure 6). However, we also observed some differentiation on PC3 that is not explained by geography of sampling, sex, or spawning date. We have also assessed several potential methodological origins, none of which explain this variation (e.g., sequencing library effects). Genetic loci that contribute strongly to this pattern are scattered across the reference genome rather than concentrated in a single genomic region, indicating that this structure is not tied to a single gene or region of the genome. Because the structure is shared across the major geographic division in the system, Boysen Dam, we infer that this genetic structure pre‐dates differentiation caused by the construction of the dam. Most puzzlingly, because there is not a spatial component to the differentiation, assuming it is biological and not methodological in origin, it is somehow maintained in sympatry, as if there are consistently two distinct spawning types present throughout the system. However, again, we observe no clear correlation with spawning time or geographic area. Future work should further investigate this pattern.

### 4.3 Management implications

Both population structure and interspecific hybridization in the Wind and Bighorn Rivers appear to be influenced by dams. Dams and diversions on the lower Wind River and upper Bighorn River prevent sauger dispersal between the Wind River and Bighorn River populations, thereby preventing any potential demographic or genetic rescue of Wind River sauger. The reservoir formed by Boysen Dam contained the highest proportion of walleye, over half of the identified hybrid individuals, and was the only location with a first‐generation hybrid. These results, in conjunction with other research on sauger × walleye hybridization and demographic effects of dams on sauger populations, further demonstrate the negative impacts of dams on riverine fishes. Additionally, this research also highlights the potential for negative effects of dams on sauger population evolutionary trajectories via decreased genetic diversity and increased susceptibility to introgression from walleye.

We identified genetic divergence between populations upstream and downstream of Boysen Dam (Figure 6), but this genetic divergence is relatively weak, likely reflecting recent restriction of movement by construction of Boysen Dam. Within the Wind River and within the Bighorn River, populations of sauger appear to be genetically homogenous. While the Wind River and Bighorn River populations can likely be managed as a single population from the perspective of genetic similarity, the lack of ongoing natural dispersal does effectively mean that these populations are separate and will continue to diverge unless connectivity is reestablished. Similarly, the lower genetic diversity in Wind River sauger populations is unlikely to increase without increases in population size or influx of individuals from other populations (though any effects of low genetic diversity on fitness have yet to be demonstrated in this system). Taken together, all of this genetic evidence points to the existence of a currently subdivided population with high levels of gene flow within the Bighorn River and within the Wind River that would likely reconnect if fish passage were possible. However, there are likely logistical challenges to any potential fish passage solutions as this would require considering not just Boysen Dam, but also other low head dams on the Bighorn River.

The risk of genetic homogenization of sauger and walleye in this system appears to be low; hybridization is infrequent enough that sauger are likely to persist as mostly genetically distinct from walleye even if a low baseline level of hybridization occurs. However, the notably higher prevalence of hybridization in Boysen Reservoir further highlights the potential for reservoirs and other impounded river sections to foster hybridization in systems where it is otherwise rare or nonexistent. While this is concerning, as reservoirs are a common feature across the sauger's native range, it also presents an opportunity for managers to target monitoring efforts. We detected very few hybrid individuals outside of reservoirs, which suggests that future hybridization monitoring programs in this system and others would benefit from prioritizing assessment of individuals within reservoirs.

Fish phenotype was not found to be a good predictor of hybrid status. Previous studies did not find hybrids in Boysen Reservoir or the Bighorn River (Bingham et al., 2012; Krueger et al., 1997), and this may be because they targeted fish that looked to be phenotypically sauger and not walleye. Future studies of hybridization will be most informative if they sample individuals with both sauger and walleye phenotypes, as most of the individuals genetically identified as hybrids and all of the intermediate hybrids were initially phenotypically identified as walleye rather than sauger (Table 2). These findings suggest that a walleye‐like phenotype might be more characteristic of some hybrids, and confirms that phenotypic identification of hybrids can be challenging.

Finally, it is important to emphasize that current hybridization dynamics do not necessarily predict future hybridization dynamics. Environmental conditions affect recruitment of sauger, walleye, and hybrids (Graeb et al., 2010), and natural or anthropogenic changes to conditions might alter the fitness landscape for parental species and hybrids (Butt et al., 2017). Changing climate or biotic conditions might also present new enhanced opportunities for hybridization; historically, hybridization has been much more common in disturbed or modified environments, and a growing body of evidence suggests that global climate change might promote or accelerate hybridization (Chunco, 2014; Muhlfeld et al., 2014). It is also possible that hybridization might lag far behind the initial introduction of walleye, as in some other systems (Mandeville et al., 2019).

## AUTHOR CONTRIBUTIONS

**William C. Rosenthal:** Data curation (equal); formal analysis (lead); investigation (lead); methodology (equal); validation (equal); visualization (lead); writing – original draft (equal); writing – review and editing (equal). **Elizabeth G. Mandeville:** Conceptualization (equal); data curation (equal); formal analysis (supporting); funding acquisition (equal); investigation (supporting); methodology (equal); writing – original draft (equal); writing – review and editing (equal). **Ashleigh M. Pilkerton:** Data curation (supporting); visualization (supporting); writing – original draft (equal); writing – review and editing (equal). **Paul C. Gerrity:** Conceptualization (equal); funding acquisition (equal); methodology (equal); resources (supporting); writing – original draft (equal); writing – review and editing (equal). **Joseph A. Skorupski:** Conceptualization (equal); funding acquisition (equal); methodology (equal); writing – original draft (equal); writing – review and editing (equal). **Annika W. Walters:** Conceptualization (equal); funding acquisition (equal); project administration (equal); writing – original draft (equal); writing – review and editing (equal). **Catherine E. Wagner:** Conceptualization (equal); funding acquisition (equal); investigation (supporting); methodology (supporting); project administration (equal); validation (equal); writing – original draft (equal); writing – review and editing (equal).

## CONFLICT OF INTEREST STATEMENT

The authors declare no conflicts of interest.

## BENEFIT‐SHARING STATEMENT

Benefits from this research accrue from our data being publicly available on the aforementioned databases. Additionally, this research has been included in a report made available to the Wyoming Game and Fish Department to inform management decisions.

## Data Availability

Genomic data have been uploaded to the NCBI Short Read Archive under BioProject ID PRJNA1128255. Scripts for analyses presented here and additional figures related to results mentioned here can be found at the following location: Rosenthal, W. C., Mandeville, E. G., Pilkerton, A. M., Gerrity, P. C., Skorupski, J. A., Walters, A. W., & Wagner, C. E. (2024). will‐rosenthal/WBH_sauger: https://doi.org/10.5281/zenodo.12192022.
